# Hysteresis in hybrid perovskite indoor photovoltaics

**DOI:** 10.1098/rsta.2021.0144

**Published:** 2022-04-18

**Authors:** Alasdair Bulloch, Shaoyang Wang, Paheli Ghosh, Lethy Krishnan Jagadamma

**Affiliations:** Energy Harvesting Research Group, SUPA, School of Physics and Astronomy, University of St Andrews, North Haugh, St Andrews KY16 9SS, UK

**Keywords:** Internet of Things, triple cation perovskites, CH_3_NH_3_PbI_3_, maximum power point tracking, ion migration

## Abstract

Halide perovskite indoor photovoltaics (PV) are a viable solution to autonomously power the billions of sensors in the huge technology field of the Internet of Things. However, there exists a knowledge gap in the hysteresis behaviour of these photovoltaic devices under indoor lighting conditions. The present work is the first experimental study dedicated to exploring the degree of hysteresis in halide perovskite indoor photovoltaic devices by carrying out both transient J–V scan and steady state maximum power point tracking (MPPT) measurements. Dependence of hysteresis on device architecture, selection of electron transporting layers and the composition of the perovskite photoactive layers were investigated. Under indoor illumination, the p-i-n MAPbI_3_-based devices show consistently high power conversion efficiency (PCE) (stabilized PCE) of greater than 30% and negligible hysteresis behaviour, whereas the n-i-p MAPbI_3_ devices show poor performance (stabilized PCE ∼ 15%) with pronounced hysteresis effect. Our study also reveals that the n-i-p triple cation perovskite devices are more promising (stabilized PCE ∼ 25%) for indoor PV compared to n-i-p MAPbI_3_ due to their suppressed ion migration effects. It was observed that the divergence of the PCE values estimated from the J–V scan measurements, and the maximum power point tracking method is higher under indoor illumination compared to 1 Sun, and hence for halide perovskite-based indoor PV, the PCE from the MPPT measurements should be prioritized over the J–V scan measurements. The results from our study suggest the following approaches for maximizing the steady state PCE from halide perovskite indoor PV: (i) select perovskite active layer composition with suppressed ion migration effects (such as Cs-containing triple cation perovskites) and (ii) for the perovskite composition such as MAPbI_3_, where the ion migration is very active, p-i-n architecture with organic charge transport layers is beneficial over the n-i-p architecture with conventional metal oxides (such as TiO_2_, SnO_2_) as charge transport layers.

This article is part of the theme issue ‘Developing resilient energy systems’.

## 1. Introduction

The Internet of Things (IoT)—connecting everyday electrical objects to the Internet—has the potential to revolutionize the manufacturing industry, transport and building sectors through real-time process monitoring and enhancing energy security. By 2025, around 75 billion objects will be connected to the IoT, and half of them will be housed indoors [1]. A recent study has shown that by incorporating IoT sensors into the building system, energy savings of up to 40% can be achieved [2]. Wireless sensors are one of the most important components constituting the IoT system. The number of objects connected to the IoT, and the number of wireless sensors needed is directly correlated. One of the main factors that limit the widespread and large-scale deployment of the IoT is the lack of a resilient powering method for the billions of wireless sensors. Even though these sensors require only micro-to milliwatts of power, usage of batteries or grid-connected electricity is not a feasible solution due to the increased installation complexity and the environmental repercussions associated with the limited lifetime of batteries [3]. Hence, developing self-powered wireless sensors that can harvest ambient energy is a promising solution. Different forms of energy are available inside buildings, including light from artificial light sources, mechanical energy due to vibration from household electrical appliances, human movement and the temperature gradient [4]. Light energy harvesting using the photovoltaic effect is the simplest and most direct method due to the high power density available inside buildings compared to the other forms of energy, and it does not require any additional intermediate steps to achieve useful electrical output. However, ‘indoor photovoltaics' (PV) for powering wireless sensors should integrate the attributes of useful electrical power output, cost-efficiency, scalability and high specific power (power per unit weight). Considering the different generation of photovoltaic technologies currently available, halide perovskite semiconductor-based PV best comprise all these characteristics [5].

Halide perovskites, represented by the chemical formula of ABX_3_, is a family of molecular semiconductors which binds both organic and inorganic components in a single molecule [6]. Within a decade of their inception, the power conversion efficiency (PCE) of halide perovskite solar cells (under 1 Sun illumination) has increased from 3.9% to 25.5% [7]. With the emergence of the IoT, the field of indoor PV is receiving a rejuvenated research interest, and halide perovskites are the leading photovoltaic materials being explored along with dye sensitized and organic PV [1,8]. Halide perovskites have already demonstrated a PCE of 40% under indoor lighting, and their capability in powering the wireless sensors was also reported recently [9–11]. Despite these promising results, there exists a lack of knowledge and understanding of the hysteresis behaviour of halide perovskite indoor PV, which determines the reliability of electrical output from these devices and their suitability as power sources for wireless sensors in the widespread deployment of the IoT.

**Figure 1.**
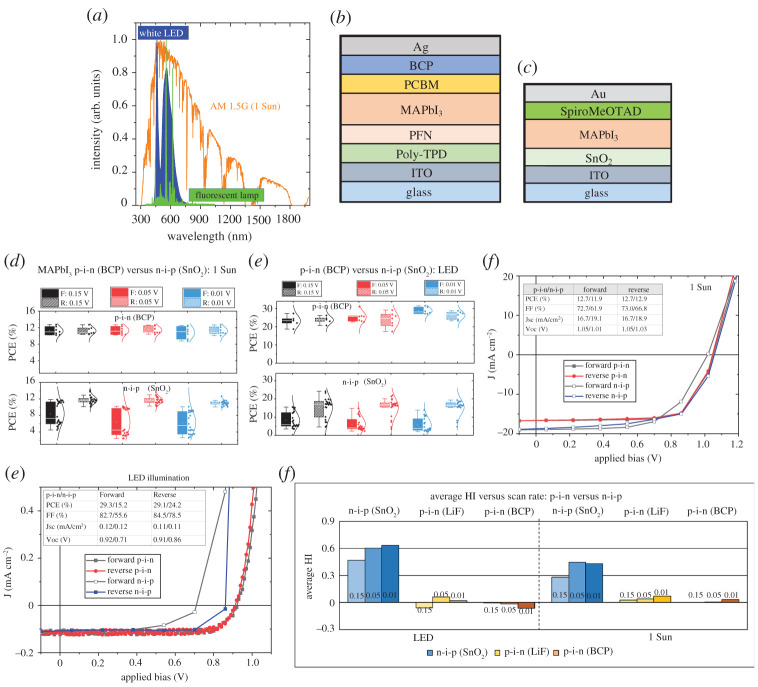
(*a*) Comparison of the spectra from the indoor light sources such as a warm white LED (at 1000 lux and irradiance of 0.3 mW cm^−2^) and CFL (at 1000 lux) with that of the 1 Sun (AM 1.5G, irradiance of 100 mW cm^−2^) illumination. (*b*) The p-i-n device architecture used in this study. (*c*) The n-i-p device architecture used in this study. (*d*) PCE values of p-i-n and n-i-p MAPbI_3_ devices as a function of scan rate under 1 Sun illumination. (*e*) PCE values of p-i-n and n-i-p devices as a function of scan rate under indoor illumination. (*f*) J–V characteristics of the champion devices under 1 Sun illumination. (*g*) J–V characteristics of the champion devices under LED illumination. (*h*) Comparison of HI for p-i-n and n-i-p devices as a function of different scan rates under two different illumination conditions. (Online version in colour.)

The hysteresis in J–V curves for halide perovskite solar cells refers to the situation where the J–V curves obtained from the forward voltage scan (short circuit to open circuit) and reverse voltage scan (open circuit to short circuit) differ considerably resulting in different PCE values depending on the scan direction, the voltage range scanned and the scan rate [12]. Overcoming the J–V hysteresis has been one of the most critical challenges during the development phase of perovskite solar cell technology [13]. Studies have established that under 1 Sun illumination, the J–V hysteresis in halide perovskite solar cells is influenced by device architecture (p-i-n versus n-i-p), the selection of interface charge-transporting layers, the composition of the perovskite photoactive layer and the measurement conditions such as scan rate, pre-bias and light soaking [12,14–17]. The most recent prevailing view is that hysteresis in perovskite solar cells is a consequence of the mobile ions and their impact on charge carrier extraction and recombination which in turn are influenced by both electrical bias and light intensity [18–20]. In indoor PV, the incident light is from artificial light sources such as white light-emitting diodes (LEDs) and compact fluorescent lamps (CFLs). These light sources differ considerably from the outdoor 1 Sun illumination in spectral range, spectral content and intensity as shown in figure 1*a*. The 1 Sun spectral range extends from 280 to 2500 nm with the irradiance of 100 mW cm^−2^, whereas the indoor artificial light sources mainly emit in the visible range and the intensity ranges from 0.05 to 0.5 mW cm^−2^ (200–2000 lux). Since the J–V hysteresis in halide perovskite PV is influenced by the electric field distribution which in turn can be modulated by the photogenerated charge carriers, it is imperative to re-visit the hysteresis issue but from the perspective of indoor PV. To the best of the authors' knowledge, this is the first report detailing the investigation of hysteresis behaviour in halide perovskite indoor PV.

In the present work, we are addressing such questions as: does halide perovskite photovoltaic devices show similar hysteresis behaviour under indoor light as well as 1 Sun illumination? If different, how is this hysteresis behaviour influenced by device architecture and selection of the active layer? What could be the possible reasons for the difference in J–V hysteresis under indoor and 1 Sun illumination? To answer these questions, we have investigated the scan rate-dependent J–V hysteresis in halide perovskite-based photovoltaic devices under both indoor illumination (warm white LED at 1000 lux, colour temperature of 2700 K and irradiance of 0.3 mW cm^−2^) and 1 Sun (AM 1.5G spectrum, irradiance of 100 mW cm^−2^) as a function of different device architectures (n-i-p versus p-i-n), three different compositions of the active layer, CH_3_NH_3_PbI_3_ (referred to as MAPbI_3_ hereafter), CsPbBr_2_I and Cs_0.05_FA_0.81_MA_0.14_PbI_2.55_Br_0.45_ (referred to as TCA hereafter, the acronym for Cs-containing triple cation perovskites) and different electron transport layers (ETLs). We also investigate the hysteresis issue in these devices using the steady state method of maximum power point tracking (MPPT), as this approach closely matches the conditions under which these devices would be operated when they are deployed in their real-life applications.

Our results reveal that p-i-n MAPbI_3_ devices with all-organic interlayers are highly promising for indoor photovoltaic applications as these devices demonstrate a steady state PCE of greater than 30% under indoor illumination, with negligible hysteresis effect. The n-i-p MAPbI_3_ devices, employing SnO_2_ or TiO_2_ as the ETL and Spiro-OMeTAD as the hole transporting layer (HTL), suffer from high hysteresis, with a demonstrated steady state PCE of only approximately 15%. However, switching the active layer from MAPbI_3_ to triple cation perovskite resulted in the corresponding n-i-p devices delivering a steady state PCE of 25% under indoor illumination, with relatively minimal hysteresis. Light intensity analysis of the open-circuit voltage (*V*_oc_) and fill factor (FF) reveals that p-i-n devices have less trap-assisted recombination losses, and the charge collection/extraction efficiency is not impeded even at low light intensity, whereas for the n-i-p devices, the charge collection is severely disrupted under low light intensity. Our study reveals the importance of verifying the PCEs of halide perovskite indoor PV obtained through conventional J–V scan measurements with that of steady state MPPT approach, since for devices suffering from hysteresis, any deviation in PCE estimated using these methods becomes pronounced under indoor lighting conditions. Based on our results, we propose to prioritize the MPPT measurements over the conventional J–V scan method for reliable PCE estimation of halide perovskite indoor PV. Moreover, to maximize the steady state output power from the halide perovskite indoor PV, the selection of perovskite active layer composition with suppressed ion migration (TCA over MAPbI_3_ in the present case) and charge transport layers with improved carrier extraction even at low light intensity (organic transport layers over the metal oxides in the present case) were found to be beneficial.

### (a) Theoretical background

The PCE (*η*) of the laboratory-based photovoltaic devices is calculated using the following relation,

1.1 $$\eta =\frac{J_{\mathrm{sc}}\times V_{\mathrm{oc}}\times \mathrm{FF}}{P_{\mathrm{in}}}100\%,$$

where *V*_oc_ is the open-circuit voltage, *J*_sc_ is the short-circuit current density and FF is the fill factor. In the case of 1 Sun illumination, the *P*_in_ is 100 mW cm^−2^, and for the indoor lighting investigated in the present study, it is 0.3 mW cm^−2^ which corresponds to a 1000 lux illuminance from a warm white LED (colour temperature of 2700 K). This illumination level of 1000 lux corresponds to the normal illuminance of indoor artificial light sources inside office buildings [21]. Previous theoretical calculations [22] have shown that the Shockley–Queisser limit for PCE of indoor PV under different artificial light sources can reach as high as 50%. This high PCE is due to the reduced thermalization and non-absorption losses through the better spectral overlap of the absorption spectra of the photoactive materials with the indoor light spectra.

When considering the difference between the PCE under indoor and outdoor illumination, the relationship of each of these photovoltaic performance parameters to that of light intensity needs to be known [23,24].

The open-circuit voltage *V*_oc_ depends on the light intensity *I* logarithmically through the following formula,

1.2 $$V_{\mathrm{oc}}=\frac{nk_{B}T}{q}\ln(I),$$

where *n* is the diode ideality factor, *k_B_* is the Boltzmann constant, *T* is the temperature and *q* is the charge of the electron.

The short-circuit current density (*J*_sc_) depends on the light intensity through the power-law shown below,

1.3 $$J_{\mathrm{sc}}\propto I^{\alpha },$$

where *α* is the recombination value. The FF depends on the light intensity indirectly through *V*_oc_, shunt and series resistance [8,25].

We used a metric of hysteresis index (HI) to compare the J–V hysteresis of the perovskite solar cells under the two different illumination conditions. There is a wide debate regarding the use of this term when comparing the optimized steady state performance properties of different solar cells, as HI measures arbitrary non-steady state values [18,26,27]. However, Pintilie and colleagues [26] have reported that systematically conducted non-steady state measurements under the influence of external parameters (two different illumination conditions, different scan rate and scan directions in the present study) can support the performance optimization of perovskite solar cells.

The HI is estimated using the following relation,

1.4 $$\mathrm{HI}=\frac{\mathrm{PC}E_{\mathrm{reverse}}-\mathrm{PC}E_{\mathrm{forward}}}{\mathrm{PC}E_{\mathrm{reverse}}}.$$

To verify our observations regarding the hysteresis of halide perovskite indoor photovoltaic devices from the J–V scan analysis, we carried out the steady state measurement of maximum power point tracking as well for the corresponding devices since the latter represents accurately the performance of the device under real operating conditions [18,28].

## 2. Results and discussion

### (a) Device architecture: p-i-n versus n-i-p

Initially, the J–V hysteresis behaviour of the most widely studied halide perovskite, CH_3_NH_3_PbI_3_ in p-i-n and n-i-p device architectures (as shown in figure 1*b*,*c*, respectively) was compared under indoor LED lighting and 1 Sun illumination. Three different voltage steps of 0.15, 0.05 and 0.01 V were considered corresponding to scan rates of approximately 0.59, 0.20 and 0.04 V s^−1^ to test the effect of both fast and slow scan rates [13]. The scan rate-dependent PCEs of the n-i-p and p-i-n MAPbI_3_-based devices under 1 Sun and indoor white LED illumination sources are shown in figure 1*d* and *e*, respectively. Under 1 Sun, p-i-n MAPbI_3_ devices demonstrate minimal hysteresis as the PCE values in forward and reverse scans are very similar as shown by the overlap of the data ranges (figure 1*d*), whereas the corresponding n-i-p devices show strong hysteresis as the reverse scan gives higher PCE values than the forward scan. For n-i-p devices, there is also a significantly greater distribution of PCE values for the forward scan compared to reverse where the values are much more consistent. However, the scan rate appears to have very little influence on the PCE variation under 1 Sun, and both the p-i-n and n-i-p champion devices show comparable PCEs of 12.7% and 12.9% (figure 1*f*). The minimal hysteresis effect in p-i-n architecture compared to n-i-p under 1 Sun illumination has been previously reported and is attributed to the lack of capacitive current and the suppression of polarization properties (if any) of the perovskite photoactive layer due to the absence of metal oxide charge transport layers in the former [15,29,30]. Compared to the organic charge-transporting layers (in the p-i-n structure here), the metal oxide/perovskite interfaces are more susceptible to charge trapping effects due to the presence of defects at the ETL/perovskite interface which can contribute to the enhanced hysteresis behaviour [31,32].

It is interesting to note that under the indoor LED lighting conditions, both the PCE and the hysteresis behaviour are entirely different compared to 1 Sun illumination. The PCE of the p-i-n-based MAPbI_3_ devices increases to 29% with negligible hysteresis, whereas the n-i-p devices not only show lower PCE (15% forward scan/24% reverse scan) but also higher hysteresis (figure 1*e*,*g*). Despite the minimal hysteresis in the case of p-i-n devices, decreasing the scan rate results in a slight increment in the hysteresis (figure 1*h*). The n-i-p devices show greater J–V hysteresis under LED than under 1 Sun which is further enhanced with a decrease in scan rate (figure 1*h*). The lower hysteresis behaviour of the p-i-n devices under both 1 Sun and indoor illumination is also verified using lithium fluoride as an electron buffer layer replacing the bathocuproine as shown in electronic supplementary material, figure S1(a). A summary of the average HI and average PCE as a function of different scan rates for both device architectures is shown in figure 1*h* and electronic supplementary material, figure S1(b), respectively, and clearly demonstrates the lower HI (less than 10%) for the p-i-n device architecture under both indoor and 1 Sun illumination. By contrast, the n-i-p devices show a very high HI of 30–40% under 1 Sun illumination which increases to 40–60% under low-intensity LED illumination conditions. The n-i-p devices not only demonstrate higher HI for slower scan rates but they also show that slowing the scan rate has a more pronounced effect on hysteresis under LED conditions as compared to 1 Sun.

To understand the contribution of each of the photovoltaic performance parameters, Voc, FF and Jsc, on the hysteresis behaviour, their variation is plotted as a function of different scan rates as shown in electronic supplementary material, figure S2. The negligible hysteresis in p-i-n architecture (electronic supplementary material, figure S2(a),(b)) makes it difficult to correlate the PCE versus scan rate behaviour to the corresponding photovoltaic performance parameters versus scan rate. However, for the n-i-p devices, the PCE versus scan rate follows the same trend as that of FF (electronic supplementary material, figure S2(c),(d)). Also, the J_sc_ of the n-i-p devices systematically decreases with a decrease in scan rate. These observations imply that upon decreasing the scan rate, the charge collection/extraction of the photogenerated charge carriers is impeded in the n-i-p device architecture considered here. A similar observation showing an increase in hysteresis and decrease in J_sc_ with a decrease in scan rate has been previously reported for n-i-p halide perovskite solar cells under 1 Sun illumination [12,13]. With the decrease in scan rate, the contribution of ion migration to the J–V characteristics increases. Ion migration can modify the electric field distribution in the active layer, introduce traps/passivate traps within the photoactive layer and form a charge extraction barrier at the interfaces leading to recombination losses and charge trapping/de-trapping [15,33–36]. The higher J–V hysteresis in the n-i-p devices compared to p-i-n implies that this charge trapping and ion migration induced surface or bulk recombination losses are relatively high in the MAPbI_3_ n-i-p devices compared to the corresponding p-i-n devices. Considering that metal oxide/perovskite interfaces in n-i-p devices are more susceptible to charge trapping effects due to the presence of defects compared to organic charge transport layer/perovskite interfaces in the p-i-n architecture, the effect of ETL selection is investigated which could help us further understand the hysteresis mechanism in n-i-p devices.

### (b) Electron transporting layer comparison

**Figure 2.**
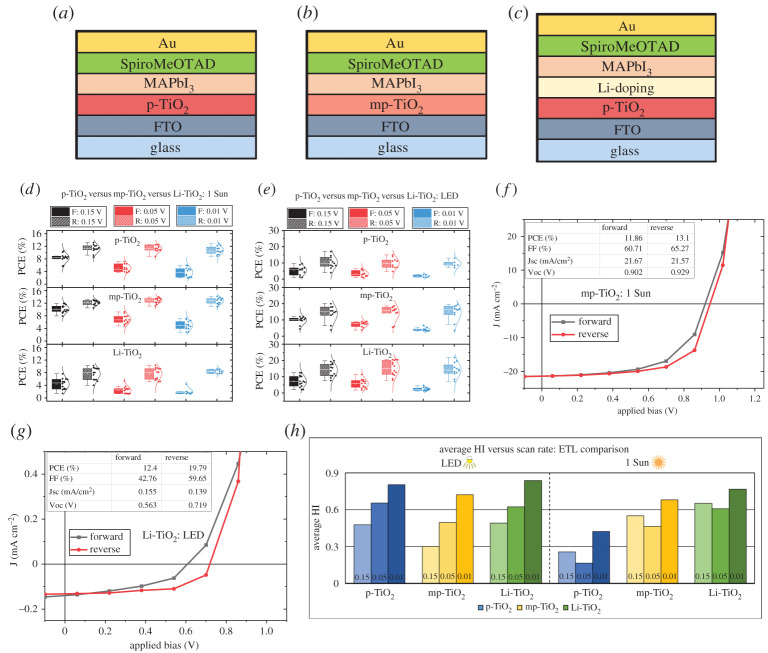
(*a*–*c*) The device architecture of the n-i-p MAPbI_3_ devices with three different types of TiO_2_; (*a*) p-TiO_2_, (*b*) mp-TiO_2_ and (*c*) Li-TiO_2_. (d) PCE values of TiO_2_ ETL-based MAPbI_3_ n-i-p devices as a function of scan rate under 1 Sun illumination. (*e*) PCE values of TiO_2_ ETL-based MAPbI_3_ n-i-p devices as a function of scan rate under LED illumination. (*f*) J–V characteristics of the champion device under 1 Sun illumination. (*g*) J–V characteristics of the champion device under indoor illumination. (*h*) HI variation as a function of scan rate for different TiO_2_-based n-i-p MAPbI_3_ devices under two different illumination conditions. (Online version in colour.)

To further verify the enhanced hysteresis of n-i-p architecture under indoor lighting conditions, the hysteresis effect was compared for n-i-p MAPbI_3_ devices using three different ETLs based on modified TiO_2_, specifically, planar TiO_2_ (p-TiO_2_), mesoporous TiO_2_ (mp-TiO_2_) and lithium-doped TiO_2_ (Li-TiO_2_) (schematic of device structures shown in figure 2*a–c*). These ETLs were selected because these are the most commonly used electron transporting layers in perovskite solar cells other than SnO_2_ [37–39]. Previously, it has been shown that Li doping in TiO_2_ can passivate the surface traps at the titania surface layer, which is one of the buried interfaces within the perovskite photoactive layer in a completed photovoltaic cell [40]. The variation in device performance as a function of scan rate under different illumination conditions using these ETLs and MAPbI_3_ as the active layer is shown in figure 2*d*,*e*. Under both illumination conditions, irrespective of the different ETLs used, for the fastest scan rate (voltage step of 0.15 V), there is a moderate amount of overlap in the PCE values in the forward and reverse scans, whereas, for the slowest scan rate (0.01 V voltage increment), there is a large mismatch between forward and reverse scan PCEs. This observation is consistent with that of SnO_2_ based n-i-p MAPbI_3_ devices as described above. Under 1 Sun, the mp-TiO_2_ ETL-based devices show a maximum PCE of 13% (figure 2*f*), while under LED illumination, Li-TiO_2_ ETL-based devices demonstrated approximately 20% PCE for the reverse scan (figure 2*g*). Under LED illumination, all the devices showed improved PCE over 1 Sun conditions with PCE values nearing 20% (figure 2*e* and electronic supplementary material, figure S3). Mesoporous and Li-doped TiO_2_ ETL-based devices show consistently improved performance under indoor LED conditions as compared to p-TiO_2._ This could be related to the better charge generation/extraction in the case of mp-TiO_2_-based devices and defect passivation due to Li doping in Li-TiO_2_, the effect of which becomes more pronounced under low-intensity indoor illumination where the number of photogenerated charge carriers is far less compared to 1 Sun. Electronic supplementary material, figure S4 and figure S5 support this inference where it shows that the improved PCE is due to enhancement in FF and Jsc. The scan rate dependence of different photovoltaic parameters, as shown in electronic supplementary material, figures S4 and S5, implies that PCE variation with scan rate mirrors that of FF and Voc under both illumination conditions and is similar to the observation from SnO_2_/MAPbI_3_ n-i-p devices. The FF is related to charge collection efficiency, and the results show that in TiO_2_ ETL-based n-i-p MAPbI_3_ devices, the FF is greatly influenced by the ion migration induced charge extraction barriers at the interfaces and trapping/de-trapping of charge carriers. This observation matches with the previous reports that the hysteresis effect mainly intervenes with the charge extracting interfaces [18,41].

Figure 2*h* and electronic supplementary material, figure S3 show the variation of HI and average PCE as a function of scan rate under the two illumination conditions. Under 1 Sun, the average HI for the intermediate scan rate (voltage step of 0.05 V) is consistently the lowest for all the TiO_2_ ETLs. All devices, irrespective of ETL used, showed increased hysteresis under LED conditions as compared to 1 Sun. The enhanced hysteresis behaviour of the n-i-p devices under indoor lighting could be due to the low intensity of the incident light. This reduced intensity would result in a smaller number of photogenerated charge carriers (compared to 1 Sun), and hence, the effect of modification of the interfaces due to ion migration or charge trapping due to defects would be more pronounced. Increased HI of halide perovskite photodiodes under low-intensity sunlight illumination has been recently reported by Zhou *et al.* [42].

### (c) Active layer composition

**Figure 3.**
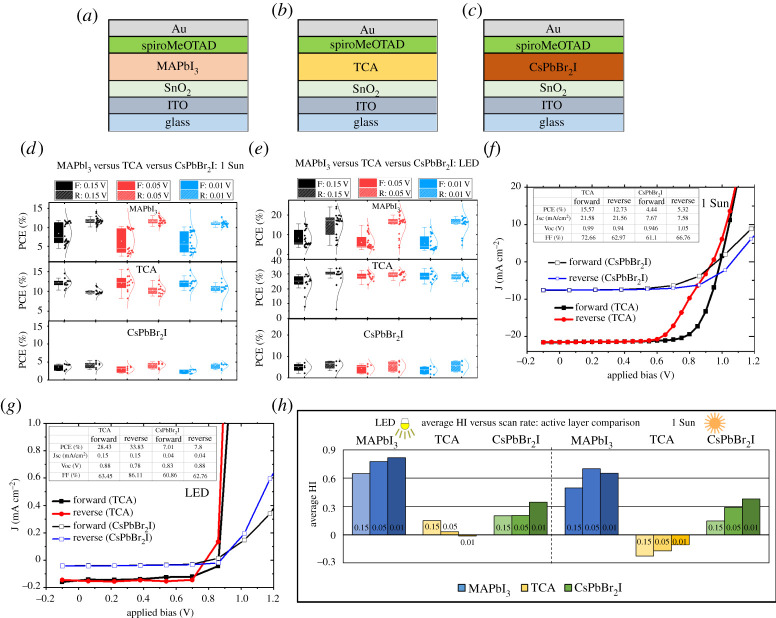
(*a*–*c*) The n-i-p device architecture with three different active layers used to investigate the hysteresis effect under different scan rates and illumination conditions. (*d*) Comparison of PCEs of devices with different active layers under 1 Sun illumination and at three different scan rates. (*e*) Comparison of PCEs of devices with different active layers under indoor LED illumination and at three different scan rates (*f*) J–V curves of the champion TCA and CsPbBr_2_I based devices under 1 Sun. (*g*) J–V curves of the champion TCA and CsPbBr_2_I based devices under indoor LED illumination. (*h*) Average HI shown against scan rate under both LED and 1 Sun illumination. (Online version in colour.)

To understand the influence of the choice of perovskite photoactive layer on the J–V hysteresis under indoor illumination, photovoltaic devices were fabricated in the n-i-p configuration using three different halide perovskites, MAPbI_3_, Cs_0.05_FA_0.81_MA_0.14_PbI_2.55_Br_0.45_ and CsPbBr_2_I. The schematic of the devices is shown in figure 3*a*–*c*. The PCE values of the corresponding devices as a function of different scan rates are shown in figure 3*d*,*e*. The TCA- and CsPbBr_2_I-based devices show lower J–V hysteresis compared to MAPbI_3_-based devices under both illumination conditions. The TCA devices showed significantly reduced hysteresis under indoor lighting compared to 1 Sun, and for both illuminations, the effect was reduced with slower scan rates resulting in better overlap of the PCE values for the forward and reverse scans. Under 1 Sun, the TCA-based solar cells demonstrate a champion PCE of 15.6% while the CsPbBr_2_I-based devices show a maximum PCE of 5.3% (figure 3*f*). All devices, irrespective of the active layer used, demonstrate better performance under LED illumination compared to that under 1 Sun (figure 3*e* and electronic supplementary material, figure S6). The TCA devices demonstrate the best PCE of 28.4% (forward scan) and 33.8% (reverse scan) under indoor LED illumination. The J–V characteristics of the champion TCA and CsPbBr_2_I devices under indoor LED illumination are shown in figure 3*g*.

The variation in HI and average PCE as a function of scan rate for different perovskite photoactive layers under the different illumination conditions is shown in figure 3*h* and electronic supplementary material, figure S6, respectively. Unlike MAPbI_3_ devices, the TCA-based devices show a decrease in HI with a decrease in scan rate under both illuminations. This signifies the suppression of ion migration in the case of TCA perovskites as previously reported [43]. The ternary cation composition of the TCA is beneficial in reducing the ion migration effects and improving the phase stability. Another important aspect is the light-induced polarization properties at the nanoscale shown by the TCA perovskite composition. This light-induced spontaneous polarization can enhance the separation of photocarriers and reduce the recombination losses without causing J–V hysteresis [43,44]. This nanoscale spontaneous polarization can also contribute to the enhanced PCE of the TCA composition under low-intensity indoor lighting. The dependence of each of the photovoltaic performance parameters of TCA and CsPbBr_2_I devices on the scan rate is shown in electronic supplementary material, figure S7 and figure S8, for the different illumination conditions. For TCA-based devices under 1 Sun and indoor illumination, the photocurrent decreases slightly with a decrease in scan rate, unlike the FF which shows slight improvement. Zhou *et al.* [19] have previously reported that the synergetic effect of electric field and light can lead to phase segregation effects in the TCA composition due to the presence of mixed halides. For the slow scan rate (with longer sweep time), the light and electric field-induced phase segregation can gradually cause the photocurrent reduction. The role of external bias-induced transient defects and trap site passivation also cannot be neglected [18,28]. For CsPbBr_2_I devices, the PCE variation with scan rate follows the same trend as that of the FF and is similar to the observation in the case of MAPbI_3_.

The results so far indicate that the J–V hysteresis behaviour of perovskite solar cells strongly depends on the illumination conditions and factors such as device architecture and the choice of perovskite photoactive layers. It has been previously reported that applying a pre-bias is effective in reducing the hysteresis effect under 1 Sun [15,26,45]. In the next section, the effect of pre-bias on J–V hysteresis under different illumination will be explored.

### (d) Effect of pre-bias

The current prevailing view of J–V hysteresis is that the phenomenon is influenced by ion migration; hence, the higher hysteresis demonstrated by the n-i-p devices probed in this study can be attributed to the modulation of the effective electric field as a result of ion migration during the J–V scans [15,33,34,45]. To test this hypothesis, a pre-bias of −1.2 V was applied to each device immediately before performing the forward and reverse scans. By applying a constant bias before scanning, the ions are forced to migrate across the active layer to the interfaces. Thus during the actual J–V scan, much of the ion migration will have already occurred, and therefore, the influence of ion migration on the dynamic J–V characteristic is suppressed. Each of the devices with the three forms of TiO_2_ as ETL which showed hysteresis during J–V measurements was tested again under 1 Sun and LED conditions. Each solar cell was first scanned without any pre-bias (as ‘normal’), followed by the application of a pre-bias of −1.2 V for 60 s and subsequent J–V measurements in forward and reverse scans. Positive pre-bias was also tested, but this decreased the PCE to around zero for both forward and reverse scans due to the ion migration direction opposing that of the charge extraction direction [26,45].

**Figure 4.**
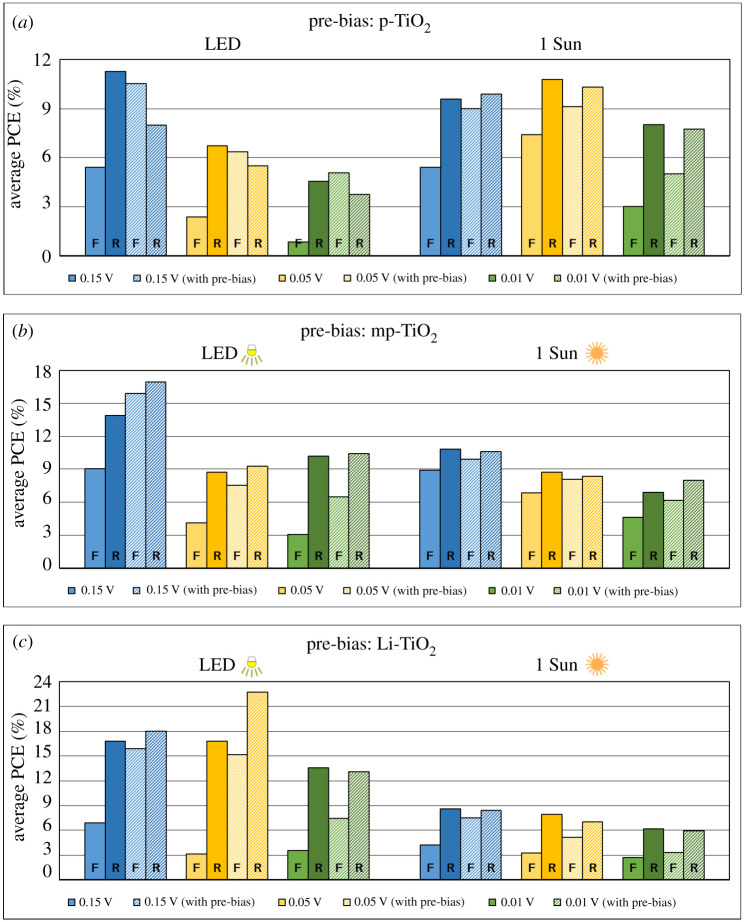
(*a*) Average PCE for p-TiO_2_ ETL based n-i-p MAPbI_3_ devices under LED and 1 Sun illumination without pre-bias (filled) and with pre-bias (shaded) for three scan rates. (*b*) Average PCE for mp-TiO_2_ ETL based n-i-p MAPbI_3_ devices under LED and 1 Sun illumination without pre-bias (filled) and with pre-bias (shaded) for three scan rates. (*c*) Average PCE for Li-TiO_2_ ETL based n-i-p MAPbI_3_ devices under LED and 1 Sun illumination without pre-bias (filled) and with pre-bias (shaded) for three scan rates. (Online version in colour.)

Figure 4*a*–*c* shows the variation in the average PCE upon applying a pre-bias in the case of the MAPbI_3_ devices with p-TiO_2_, mp-TiO_2_ and Li-TiO_2_ as ETLs, respectively. Applying a pre-bias successfully reduced the extent of hysteresis at all scan rates and under both lighting conditions. In some instances, the average PCE was observed to have increased, and the direction of the average hysteresis was reversed. Previously, it has been reported that the type of hysteresis (normal or inverted) depends on the composition of the active layer, the device architecture, different optimization processes and measurement conditions [26,46]. Since in the present case, all the devices have the same active layer (MAPbI_3_), the switching of the direction of hysteresis could be attributed to the choice of ETLs. Pre-biasing the devices had a more profound effect on reducing the J–V hysteresis under LED illumination conditions compared to 1 Sun. This could be related to the lower number of photogenerated charge carriers due to the low intensity of the indoor LED illumination, and hence, the pre-biasing is more effective in extracting the trapped/affected charge carriers under the ionic movement. Under LED conditions, Li-TiO_2_ shows the maximum reduction in hysteresis of all three types of devices. This is most likely due to the higher charge trapping and ion migration in the Li-TiO_2_ ETL compared to other TiO_2_-based ETLs. Though Li-ions are effective in passivating the defects in the titania ETL, under the influence of electric field (during the measurement), the Li^+^ ion migration also cannot be ruled out. Hence, the application of a pre-bias may effectively result in suppression of the Li^+^ ion migration along with the other ions such as iodide and their vacancies from the active layer, thus lowering the J–V hysteresis. In general, a faster scan rate results in a greater reduction in hysteresis which may allude to the effect of pre-bias reducing over time. The slower the scan rate, the more time is required to scan a device. Over the course of these tens of seconds, the suppression of ion migration induced by the pre-bias may have diminished. In electronic supplementary material, figures S9–S14, the influence of pre-bias on the different photovoltaic properties of MAPbI_3_ devices with different ETLs is shown. It is evident that for all three types of devices, the reduction in deviation of PCE between the forward and reverse scans follows the trend of FF and Voc which indicates that pre-biasing enhances the photogenerated charge carrier extraction. In summary, the application of a pre-bias to the devices before the J–V scan successfully reduces the hysteresis under both lighting conditions.

So far, the hysteresis investigation of halide perovskite indoor PV using the J–V scan analysis method has revealed the following:

(i) The p-i-n MAPbI_3_ devices showed the least J–V hysteresis under indoor and 1 Sun illumination.
(ii) For the n-i-p MAPbI_3_ devices, the J–V hysteresis effect is enhanced under indoor lighting compared to 1 Sun.
(iii) Keeping the n-i-p architecture but switching the perovskite active layer composition from MAPbI_3_ to triple cation TCA drastically reduces the J–V hysteresis under indoor lighting.
(iv) Pre-biasing the n-i-p MAPbI_3_ devices reduces the J–V hysteresis with the diminution effect prominent under indoor lighting.

In the following section, the hysteresis in these PV devices is investigated using the steady state measurement approach of MPPT.

### (e) Hysteresis investigation through maximum power point tracking

**Figure 5.**
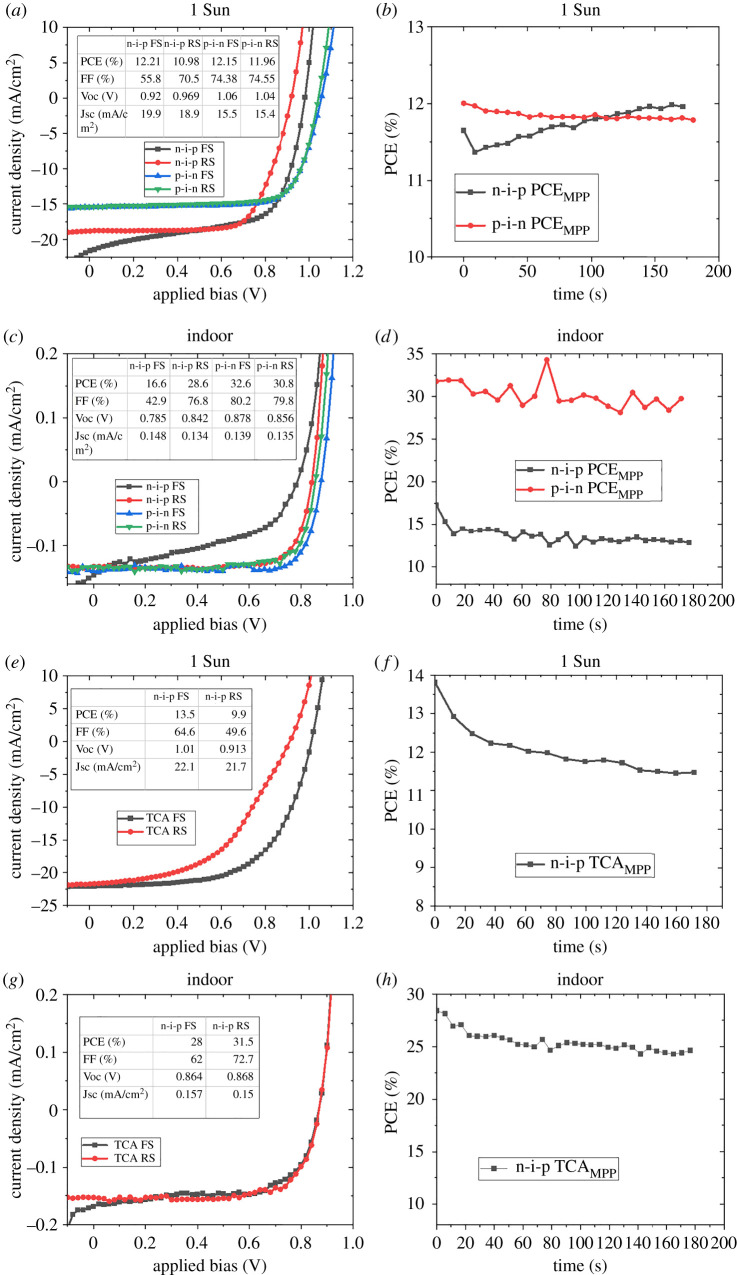
(*a*) Comparison of the J–V curves for the n-i-p and p-i-n MAPbI_3_ devices under 1 Sun. (*b*) Comparison of the MPPT curves for the n-i-p and p-i-n MAPbI_3_ devices under 1 Sun. (*c*) Comparison of the J–V curves for the n-i-p and p-i-n MAPbI_3_ devices under indoor illumination. (*d*) Comparison of the MPPT curves for the n-i-p and p-i-n MAPbI_3_ devices under indoor illumination. (*e*) J–V curves for the n-i-p TCA devices under 1 Sun. (*f*) MPPT PCE for the n-i-p TCA devices under 1 Sun. (*g*) J–V curves for the n-i-p TCA devices under indoor illumination (*h*) MPPT PCE for the n-i-p TCA devices under indoor illumination. (Online version in colour).

Since the hysteresis behaviour estimated through J–V scan analysis is more dependent on measurement conditions than the actual device and material properties, hysteresis properties of the MAPbI_3_ (both n-i-p and p-i-n) and n-i-p TCA photovoltaic devices were studied using the maximum power point tracking method. Figure 5*a* shows the J–V curves of the typical n-i-p and p-i-n MAPbI_3_ photovoltaic devices under 1 Sun illumination. Compared to the p-i-n devices, the n-i-p devices suffer from higher J–V hysteresis as already discussed in §2*a*. For the p-i-n devices, the forward and reverse scan PCEs show substantial overlap, whereas a large deviation is shown by the corresponding n-i-p devices. The PCE obtained from the maximum power point tracking of the corresponding devices is shown in figure 5*b*. Under 1 Sun illumination, the PCE of the p-i-n devices stabilizes quickly (less than 10 s) unlike the n-i-p devices, where it takes longer (several tens of seconds, ∼100 s) for the PCE to stabilize. The longer time taken to stabilize the photocurrent in the n-i-p devices can be considered as an indication of ionic migration-induced electric field effects on the photogenerated carriers and their intervention in the charge extraction at the interfaces. For the p-i-n devices, the efficiencies estimated from the J–V curves can be corroborated with those obtained from the MPPT measurements, thus implying the high quality of interfaces where the charge extraction is not being modulated by ionic migration and low surface charge or bulk recombination within the perovskite solar cell [18,41].

Under indoor illumination, as shown in figure 5*c,d*, the hysteresis in J–V curves is enhanced for n-i-p MAPbI_3_ devices as explained in §2*a*. From the J–V scan measurements, the n-i-p MAPbI_3_ devices show PCEs of 16.6% (forward scan) and 28.6% (reverse scan), whereas the p-i-n MAPbI_3_ devices show higher and more closely matching PCEs of 30.8% (forward scan) and 32.6% (reverse scan). However, upon MPPT measurement, the corresponding n-i-p and p-i-n MAPbI_3_ devices demonstrated a PCE of 15% and 32%, respectively. It is interesting to note that the PCE estimated from the J–V scan closely matches that from the MPPT measurements for the p-i-n devices, under indoor lighting, and it indicates the reliable performance of the corresponding devices, undistorted by ionic field induced modifications at the interface. The slight fluctuation in the PCE measured in the MPPT can be attributed to the noise from the indoor light source where no special driving circuitry is being used to minimize the temporal variations in intensity. In the case of n-i-p MAPbI_3_ devices, the deviation in the PCE estimated from the MPPT method and J–V scan is substantial (28% in reverse scan versus 15% in MPPT). This large divergence between the steady state efficiency and the J–V scan efficiency in the n-i-p MAPbI_3_ devices is an indication of inferior quality of the interface with strong ionic modulation effects. In the case of metal oxide charge transport layers (SnO_2_ and TiO_2_ in the n-i-p devices considered here), defects such as oxygen vacancies can cause charge trapping/de-trapping and accumulation of ionic defects and are therefore more prone to transient modulation of charge extraction properties at the interface. The poor quality of the interfaces and any charge trapping will be more pronounced under the low-intensity illumination due to the lower number of photogenerated carriers [18,41,47]. However, it is interesting to note that both the p-i-n and n-i-p MAPbI_3_ devices show very similar rapid stabilization behaviour for the PCE measurement under indoor light conditions using the MPPT method (figure 5*d*).

In figure 5*e*–*h*, a comparison of the PCE estimated from the J–V curves and the MPPT is given for the n-i-p TCA devices under indoor and 1 Sun illumination. As shown in figure 5*f*, the n-i-p TCA devices require a longer duration for the PCE to stabilize under 1 Sun illumination as in the case of n-i-p MAPbI_3_ devices (figure 5*b*). For indoor illumination, contrary to what has been observed for n-i-p MAPbI_3_ devices, the J–V hysteresis drastically reduces (28% forward scan and 31% reverse scan), and the MPPT shows a stabilized PCE of 25%. It is important to notice that the stabilized PCE is lower than that shown by J–V scan measurements. However, the observed difference between the PCE estimated from the J–V characteristics and MPPT is lower (31% from J–V scan and 25% from the MPPT) in n-i-p TCA devices compared to that of n-i-p MAPbI_3_ devices (28% in reverse scan versus 15% in MPPT). It is worth noting that under indoor lighting, n-i-p TCA devices also show rapid stabilization of PCE (figure 5*h*).

Since the n-i-p MAPbI_3_ devices showed the largest deviation in PCE estimated from the J–V scan and the MPPT measurements under indoor illumination, the effect of pre-bias is explored in these devices. As shown in electronic supplementary material, figures S15 (a),(c), pre-bias reduces the J–V hysteresis under both 1 Sun and indoor illumination, with the improvement in PCE being higher for the indoor illumination condition. Pre-biasing of these devices enabled the faster stabilization of output power in the MPPT measurements under 1 Sun illumination (electronic supplementary material, figure S15 (b)). However, under indoor illumination, though pre-biasing improves the initial PCE recorded (from 14% to 24%), it shows a steady decline with time and stabilization time is prolonged (electronic supplementary material, figure S15(d)). The steady state PCE is not achieved for the pre-biased n-i-p MAPbI_3_ devices even after 10 min under indoor illumination. This observation underlines the necessity of verifying the efficiency enhancement through pre-biasing with the corresponding MPPT measurements. Exploring the electromigration of ions using the hold voltage for MPPT measurement under different illumination conditions is necessary to shed further light on this observation [14,47].

Thus, the comparison of the hysteresis behaviour of the selected halide perovskite *indoor* photovoltaic devices studied using the J–V scan method, and the MPPT approach reveals the following:

(i) The p-i-n MAPbI_3_-based devices show consistently high PCE of greater than 30% and negligible hysteresis behaviour in both J–V scan and MPPT measurements.
(ii) The n-i-p MAPbI_3_ devices show the largest deviation in the PCE estimated from the J–V scan and MPPT measurements.
(iii) Compared to n-i-p MAPbI_3_, the n-i-p TCA devices are more promising for indoor PV with stabilized PCE of approximately 25%.
(iv) The MPPT PCE stabilization is rapid under indoor lighting conditions compared to 1 Sun illumination and is hardly influenced by device architecture or the composition of the active layer considered.
(v) The divergence of the PCE values estimated from the J–V scan measurements and the MPPT method is higher under indoor illumination conditions compared to 1 Sun conditions.

In the following section, the possible reasons for these observations are discussed.

## 3. Discussion

**Figure 6.**
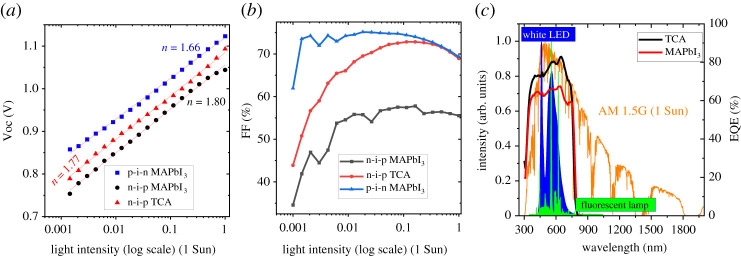
(*a*) Light intensity dependence of Voc for MAPbI_3_ (n-i-p and p-i-n) and TCA devices. (*b*) Light intensity dependence of FF for MAPbI_3_ (n-i-p and p-i-n) and TCA devices. (*c*) EQE spectra of the n-i-p TCA and MAPbI_3_ devices along with the indoor and 1 Sun illumination spectra. (Online version in colour).

To verify the role of traps and the charge extraction barrier at the interfaces, light intensity dependence of Voc and FF was measured for the n-i-p (MAPbI_3_ and TCA) and p-i-n (MAPbI_3_) devices as shown in figure 6*a*,*b*. The p-i-n MAPbI_3_ devices show the lowest slope (1.66 kT/q) for the Voc versus light intensity compared to n-i-p architecture MAPbI_3_ or TCA devices (approx. 1.8 kT/q), implying relatively lower trap-assisted recombination in the p-i-n devices. Since under real operating conditions (which is probed via MPPT), photovoltaic devices operate close to FF, its variation with light intensity is highly relevant to understand the variation in charge extraction/collection properties as a function of light intensity. As shown in figure 6*b* for both n-i-p architecture devices, the FF drops drastically with the decrease in light intensity implying the poor charge extraction at the interfaces and the associated higher trap-assisted recombination losses as previously shown [41]. In the n-i-p devices, the illumination front surface is the SnO_2_ ETL/perovskite interface, and the oxygen vacancies in the metal oxide films can also induce electrode polarization and traps in addition to any charge accumulation due to ion/vacancy migration from the perovskite active layer [48]. Previously, Weber *et al.* [47] have shown that the perovskite/Spiro-OMeTAD HTL interface can also contribute to the hysteresis behaviour as a reversible chemical reaction or complexation of the iodide with the positively charged Spiro-OMeTAD. Even though the n-i-p TCA devices also show comparable trap-assisted recombination (from figure 6*a*) similar to that of MAPbI_3_, the higher PCE and less hysteresis of the TCA devices especially under indoor illumination can be attributed to the higher FF, enhanced EQE (figure 6*c*), higher bandgap (1.59 versus 1.56 eV of MAPbI_3_) and the optimized stoichiometry to suppress the ion migration [43].

Previous reports have shown that ion migration activation energy can be reduced to one-sixth when the incident light intensity increases from 0.05 mW cm^−2^ to 20 mW cm^−2^, resulting in enhanced ion migration with higher light intensity [20,49,50]. Fundamentally, ion migration is a defect mediated hopping process, and hence, its rate will depend on the density and position of the adjacent vacancies. The exact mechanism of photo-induced ion migration is not yet clear. However, theoretical and experimental studies have shown that this effect is linked to (i) light-induced formation of defects such as interstitials/vacancies creating more hopping pathways, (ii) modifying the binding between MA^+^ and the PbI_6_ octahedron frame by illumination, and (iii) weakening of the hydrogen bonding by photocarriers [49,51]. Recent reports by Kim *et al.* [50] and Marshall *et al.* [52] attribute the origin of this effect to the photoexcited electron-hole pair formation, followed by the interaction of holes with the iodide ions to form iodine vacancies (neutral iodine interstitials). This argument is justified by factors such as iodine partly contributing to the valence band formation, permitting this reaction to be quick, and the size of the neutral iodine atoms (1.5 Å versus 2.1 Å) is smaller than iodide ions. With the increase in light intensity, the increased number of holes generated can quickly interact with iodine sub-lattice, thus facilitating the ion migration. This low ion migration activation energy under 1 Sun illumination can account for the longer duration required for the PCE stabilization in n-i-p devices. The high activation energy required for ion migration under indoor illumination can suppress the modulation of the electric field distribution inside the perovskite photovoltaic devices, thus enabling a rapid PCE stabilization during MPPT measurements.

In the present investigation, in addition to the difference in the illumination intensity, the incident spectra are also different. The indoor illumination from the warm white LED is in the visible region, and the 1 Sun illumination extends from ultraviolet (UV) to near infrared (IR). Kim *et al.* [50] have reported that light-induced ion migration effects occur if the incident photon energy is above the bandgap of the perovskite absorber material. Since in both types of illumination considered here, the photons have energy greater than the bandgap energy of MAPbI_3_ (1.56 eV) and TCA (1.59 eV), it is possible for photo-induced ion migration to set in. However, because of the three orders of magnitude higher intensity of the 1 Sun illumination compared to indoor white LED, the photo-enhanced ion migration will be more prominent under 1 Sun as evidenced by the longer time required to stabilize the output power. Because of the presence of the IR component in the 1 Sun spectra, the influence of phonons on the ion migration also cannot be ruled out completely. A detailed investigation of ion migration under different light spectra and at different spectral ranges (UV, Visible and IR) as a function of light intensity would be required to unravel the ion migration due to different illumination types and deserves further research in the future.

## 4. Conclusion

Our investigation of the hysteresis behaviour of halide perovskites indoor photovoltaic devices using the methods of J–V scan and steady state MPPT revealed that p-i-n MAPbI_3_ devices using all-organic transport layers are highly promising for indoor light harvesting with consistently high PCE (greater than 30%) and negligible hysteresis effect. However, the corresponding MAPbI_3_ devices based on n-i-p architecture using metal oxide charge-transporting layers show a larger deviation in efficiency estimated from the J–V scan and MPPT method (approx. 2% deviation for p-i-n and 10% deviation for n-i-p architecture). This discrepancy in efficiency estimated from the J–V scan and MPPT is more pronounced under indoor lighting conditions for the n-i-p MAPbI_3_ devices (approx. 2% deviation for p-i-n and 90% deviation for n-i-p architecture). Compared to MAPbI_3_ devices in the n-i-p configuration, the corresponding triple cation perovskite devices are promising for indoor PV with higher PCE (MPPT PCE of 25% versus 15%) and lower hysteresis. Moreover, the performance estimation of halide perovskite-based indoor photovoltaic devices can lead to erroneous conclusions if reliant only on their traditional J–V scan-based PCE. Based on the results from our study, we propose to prioritize electrical characterization of halide perovskite indoor PV using the steady state MPPT measurements over that of conventional J–V scan to ensure their reliable deployment in real-life applications. In addition, the insights developed from the present study demand two important device design strategies for halide perovskite indoor PV, namely the choice p-i-n architecture with organic charge transport layers and the selection of photoactive layers with composition tuning to suppress the ion migration such as in triple cation halide perovskites. The combination of metal oxide charge transport layer and a perovskite photoactive layer (such as MAPbI_3_) where the ion migration is not deliberately suppressed is not ideal for realizing efficient indoor PV.

## Data Availability

The research data underpinning this publication can be accessed at doi:10.17630/a74c0f30-2586-4ec9-9b81-2c0e4f684221. The data are provided in electronic supplementary material [53].
